# The protective effect of geniposide on human neuroblastoma cells in the presence of formaldehyde

**DOI:** 10.1186/1472-6882-13-152

**Published:** 2013-07-01

**Authors:** Ping Sun, Jin-yan Chen, Jiao Li, Meng-ru Sun, Wei-chuan Mo, Kai-li Liu, Yan-yan Meng, Ying Liu, Feng Wang, Rong-qiao He, Qian Hua

**Affiliations:** 1 School of Preclinical Medicine, Beijing University of Chinese Medicine, 11 Bei San Huan Dong Road, Beijing, Chaoyang District, 100029, China; 2 State Key Laboratory of Brain and Cognitive Science, Institute of Biophysics, Chinese Academy of Sciences, 15 Datun Road, Beijing, Chaoyang District, 100101, China; 3 School of Chinese Materia Medica, Beijing University of Chinese Medicine, 11 Bei San Huan Dong Road, Beijing, Chaoyang District, 100029, China

**Keywords:** Formaldehyde impairment, Geniposide, Neuroprotection

## Abstract

**Background:**

Formaldehyde can induce misfolding and aggregation of Tau protein and β amyloid protein, which are characteristic pathological features of Alzheimer’s disease (AD). An increase in endogenous formaldehyde concentration in the brain is closely related to dementia in aging people. Therefore, the discovery of effective drugs to counteract the adverse impact of formaldehyde on neuronal cells is beneficial for the development of appropriate treatments for age-associated cognitive decline.

**Methods:**

In this study, we assessed the neuroprotective properties of TongLuoJiuNao (TLJN), a traditional Chinese medicine preparation, against formaldehyde stress in human neuroblastoma cells (SH-SY5Y cell line). The effect of TLJN and its main ingredients (geniposide and ginsenoside Rg1) on cell viability, apoptosis, intracellular antioxidant activity and the expression of apoptotic-related genes in the presence of formaldehyde were monitored.

**Results:**

Cell counting studies showed that in the presence of TLJN, the viability of formaldehyde-treated SH-SY5Y cells significantly recovered. Laser scanning confocal microscopy revealed that the morphology of formaldehyde-injured cells was rescued by TLJN and geniposide, an effective ingredient of TLJN. Moreover, the inhibitory effect of geniposide on formaldehyde-induced apoptosis was dose-dependent. The activity of intracellular antioxidants (superoxide dismutase and glutathione peroxidase) increased, as did mRNA and protein levels of the antiapoptotic gene *Bcl-2* after the addition of geniposide. In contrast, the expression of the apoptotic-related gene - *P53*, apoptotic executer - *caspase 3* and apoptotic initiator - *caspase 9* were downregulated after geniposide treatment.

**Conclusions:**

Our results indicate that geniposide can protect SH-SY5Y cells against formaldehyde stress through modulating the expression of *Bcl-2*, *P53*, *caspase 3* and *caspase 9,* and by increasing the activity of intracellular superoxide dismutase and glutathione peroxidase.

## Background

Reactive aldehydes are generated by several endogenous and exogenous processes in biological systems. They target cell membranes, induce lipid peroxidation, mitochondrial dysfunction and oxidative stress, and eventually lead to cell apoptosis [1]. Reactive aldehydes are associated with the etiology of several neurological and psychiatric disorders including chronic alcohol abuse, vascular dementia and Alzheimer’s disease (AD) [2-4].

Formaldehyde is one of the reactive aldehydes with important clinical implications. He et al. summarized that formaldehyde can influence nerve function and that it is linked to the development of psychiatric disorders [5]. An epidemiological study has shown that histology technicians and workers who are chronically exposed to formaldehyde exhibit a marked decline in cognitive ability [6]. Formaldehyde concentrations in the hippocampus and urine of AD patients are significantly higher than those of healthy aged individuals [7]. The activity of mitochondrial aldehyde dehydrogenases, the main metabolic enzyme of formaldehyde, is significantly increased in the putamen of AD patients [1]. Furthermore, formaldehyde induces the hyperphosphorylation and aggregation of neuronal Tau protein [8] and aggregation of Tau protein *in vitro* and *in vivo*[9-11]. These misfolded amorphous deposits are able to induce apoptosis of human neuroblastoma cells (SH-SY5Y cell line) and primary rat hippocampal cells [9]. Formaldehyde can also promote the misfolding, oligomerization, and fibrillogenesis of β amyloid protein (Aβ) [12]. Therefore, formaldehyde is closely related to age-associated dementia. Recently, the discovery of effective drugs to counteract the adverse impacts of formaldehyde on the central nervous system and promoting the survival of neuronal cells, has become of great interest to many neuropharmacologists.

TongLuoJiuNao (TLJN), a traditional Chinese medicine (TCM), is clinically efficacious in the treatment of ischemic cerebral stroke and dementia (Chinese SFDA: 2004 L01620). It has been reported that TLJN can protect brain tissue against ischemia after the induction of middle cerebral artery occlusion in rats, by reducing not only infarct volume but also penumbra size [13], and alleviate the damage to ischemia/reperfusion-injured neurons [14]. Moreover, TLJN can improve learning and memory ability, promote the degradation of Aβ, and clear amyloid plaques from the AD rat brain [15]. The main ingredients of TLJN are geniposide and ginsenoside Rg1 [14]. Ginsenoside Rg1 has neuroprotective effects in mesencephalic dopaminergic cells stressed with glutamate [16], while geniposide can protect PC12 cells from hydrogen peroxide-induced cell death via its involvement in the PI3K signaling pathway and its ability to activate the glucagon-like peptide 1 receptor (GLP-1R) [15]. Geniposide is also able to regulate insulin secretion through activating GLP-1R [16]. Thus, TLJN (geniposide and ginsenoside Rg1) is likely to be an appropriate drug for the treatment of dementia. However, whether it has any protective effects on reactive aldehyde-treated neuronal cells, in particular formaldehyde-stressed neurons, remains unclear.

Direct addition of formaldehyde to the cell culture medium is a common cell culture model used in studies on aldehyde toxicity [15]. Here, we examined the neuroprotective effects of TLJN and its main ingredients, geniposide and ginsenoside Rg1, on formaldehyde-stressed SH-SY5Y cells.

## Methods

### Materials

**Figure 1:**
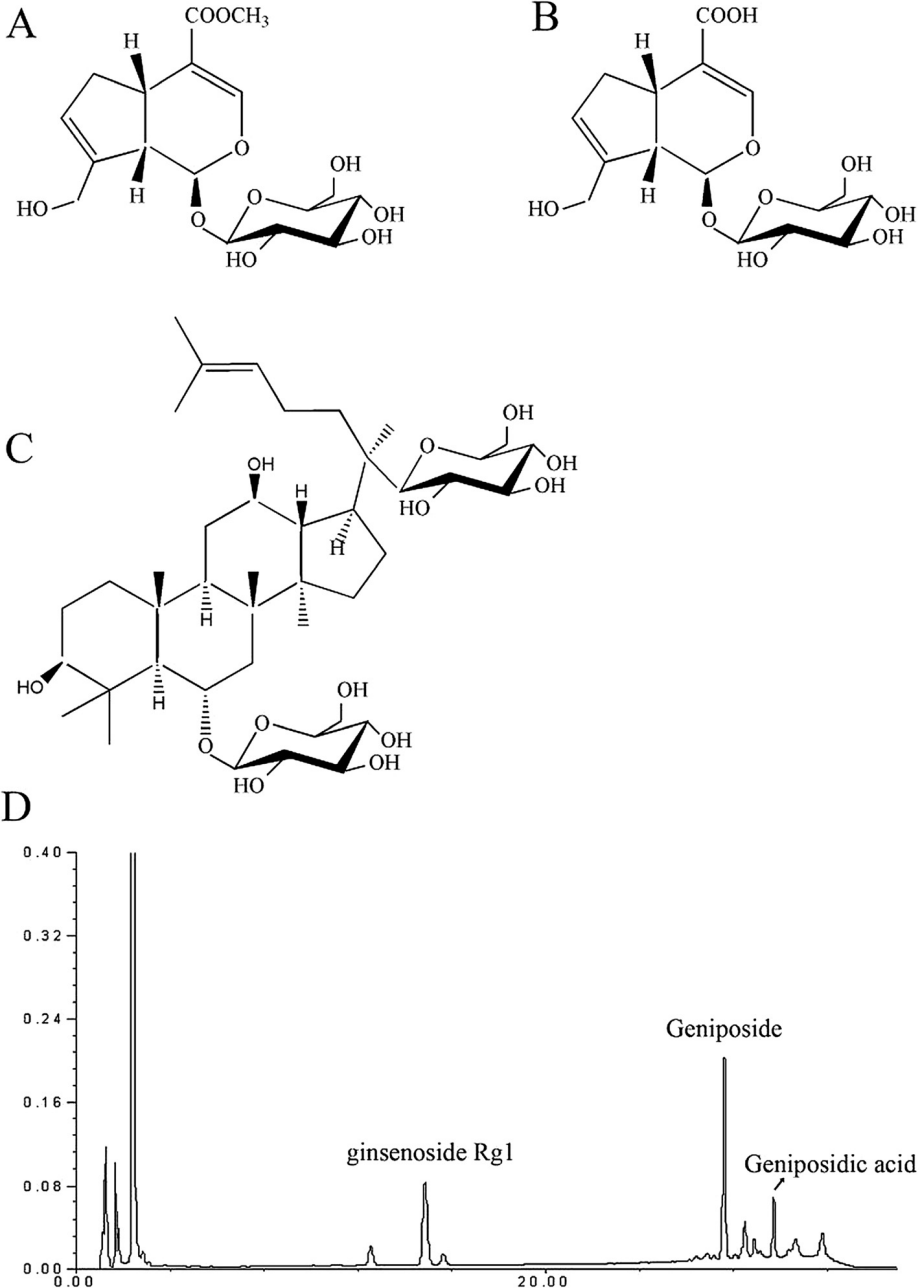
**Chemical structures and HPLC analysis of TLJN. A**. Geniposide (C_16_H_24_O_10_, molecular weight 388 Da); **B**. Geniposidic acid (C_16_H_22_O_10_, molecular weight 374 Da); **C**. Ginsenoside Rg1 (C_42_H_72_O_14_, molecular weight 800 Da); **D**. HPLC analysis of TLJN.

TLJN was provided by Kangyuan Pharmaceutical Engineering Co. Ltd (Beijing, China). The concentrations of geniposide (4.95 mg/mL) and ginsenoside Rg1 (1.02 mg/mL) in TLJN were determined by high performance liquid chromatography (HPLC; Figure 1) [14]. The purified geniposide and ginsenoside Rg1 were from the National Institutes for Food and Drug Control (Beijing, China). The cell counting Kit-8 (CCK-8) for measurement of cell viability was from Dojindo Co. (Kumamoto, Japan). Hoechst33258 was from Sigma and Alexa Fluor 488 Phalloidin was from Invitrogen (Carlsbad, CA, USA). The primary antibody against *Bcl-2* was purchased from Cell Signaling Technology (Waltham, MA, USA) and the β-actin antibody was from Beyotime Institute of Biotechnology (Jiangsu, China). The enhanced chemiluminescence (ECL) substrate was from Pierce (Boston, MA, USA) and polyvinylidene difluoride (PVDF) membrane was purchased from Millipore (Billerica, MA, USA). The annexin V apoptosis detection kit, total superoxide dismutase (SOD) assay kit with WST-1, cellular glutathione peroxidase (GSHPx) assay kit and catalase assay kit were from the Beyotime Institute of Biotechnology (Jiangsu, China). The Taq DNA Polymerase and TansStartTM Green qPCR SuperMix were purchased from Transgen Inc. (Beijing, China). The primers for *Bcl*-2, *P53*, *caspase 3*, *caspase 9*, *β-actin* and *18 s RNA* were designed and synthesized by Shanghai Sangon Biotech Co. Ltd (Shanghai, China).

### Cell culture

SH-SY5Y cells were grown in Dulbecco’s modified eagle medium (DMEM) containing 10% (v/v) fetal bovine serum (Invitrogen) and maintained in 5% (v/v) CO_2_/90% (v/v) humid air at 37°C. Cells were plated onto 96-well culture plates at a density of 1 × 10^4^ cells/well for cytotoxicity assays or 60-mm culture dishes at a density of 1 × 10^6^ cells/well for western blotting, RT-PCR, flow cytometry, and total SOD detection. After 24 h, the culture medium was replaced with serum-free DMEM supplemented with 0.12 mM formaldehyde, and different concentrations of drugs were used to treat cells as indicated in the results.

### Cell viability assay

The viability of cells was evaluated using the CCK-8 kit. Briefly, 10 μL of the CCK-8 solution was added to each well of the 96-well plate and left to incubate for 4 h in the incubator at 37°C. According to the manufacturer, simultaneous absorbance readings were measured at 450 and 630 nm using a microplate reader. The difference between the two readings was used in the final analysis.

### Cell morphology analysis

SH-SY5Y cells (plated at 3 × 10^5^) were grown in DMEM containing 10% (v/v) fetal bovine serum in 5% (v/v) CO_2_/90% (v/v) humid air at 37°C. After 12 h of plating, the culture medium was replaced with serum-free DMEM in the presence of 0.12 mM formaldehyde supplemented with 100 μM geniposide, 10 μM ginsenoside Rg1 or TLJN, which consisted of 100 μM geniposide and 10 μM ginsenoside Rg1. At different time intervals (6, 12, and 24 h), cells were fixed with 4% (w/v) paraformaldehyde. Hoechst33258 was applied to label the nuclei and F-actin was labeled with phalloidin. All images were obtained by laser scanning confocal microscopy (LSCM; Olympus FV1000, Tokyo, Japan).

### Analysis of apoptosis

Analysis of apoptosis was carried out by flow cytometry using the annexin V apoptosis detection kit. Cells were harvested, counted, and resuspended in 1 x binding buffer at a concentration of 1 × 10^6^ cells/mL. The cell suspension (100 μL) was transferred to a 5 mL culture tube and 5 μL of Annexin V-FITC was added. The suspension was gently mixed and incubated for 10 min at room temperature in the dark. Propidium iodide (PI) was added (10 μL) and samples were analyzed by flow cytometry (FACSCalibur, BD Biosciences, San Jose, CA, USA) within 1 h.

### Western blotting

Western blotting was carried out as previously described [17]. SH-SY5Y cell lysates were prepared and 50 μg of protein was loaded on a 12% (w/v) sodium dodecylsulfate-polyacrylamide gel. The separated proteins were transferred to PVDF membrane and the membrane was hybridized with an anti-*Bcl-2* monoclonal antibody (1:1000 dilution) or anti-β-actin monoclonal antibody (1:5000 dilution). Horse radish peroxidase-conjugated anti-rabbit/mouse IgG was used as the secondary antibody (1:5000 dilution).

### RT-PCR analysis

The expression of the apoptotic-related genes was detected by RT-PCR. Total RNA was extracted from SH-SY5Y cells using Trizol reagent (Invitrogen). The integrity of RNA was examined by identifying intact 18 s and 28 s ribosomal RNA bands upon agarose gel electrophoresis. The purity and quantity were estimated by measuring the absorbance at 260/280 nm spectrophotometrically. RNA was reverse-transcribed into cDNA using M-MLV reverse transcriptase (Transgen Inc.). Quantitative real time PCR was performed using TansStartTM Green qPCR SuperMix (UDG) (Transgen Inc.) according to the following reaction conditions: 50°C 2 min (UDG Incubation), 95°C 10 min (UDG Inactivation), 40–45 cycles: 5 sec 95°C, 15 sec 55°C (according to different primers), and 10 sec 72°C. The collected data were analyzed using Rotor-Gene 6000 Series Software 1.7 (Qiagen, Valencia, CA, USA). Normal RT-PCR was performed using Taq DNA Polymerase (Transgen Inc.) and detected by gel electrophoresis. The PCR primers were designed by Primerpremier 5.0 software and synthesized by Shanghai Sangon Inc. (Shanghai, China). The primers for *Bcl-2* were (TM: 55°C), forward: 5′-tgtgtggagagcgtcaacag-3′ and reverse: 5′-cagacatgcacctacccagc-3′; for *18 s RNA* (TM: 55°C), forward: 5′-cagccacccgagattgagca-3′ and reverse: 5′-tagtagcgacgggcggtgtg-3′; for *P53* (TM: 60°C), forward: 5′-acagctttgaggtgcgtgttt -3′ and reverse: 5′- ccctttcttgcggagattctct-3′; for *caspase 3* (TM: 53°C), forward: 5′-gaaattgtggaattgatgcgtga-3′ and reverse: 5′-ctacaacgatcccctctgaaaaa-3′; for *caspase 9* (TM: 53°C), forward: 5′-cttcgtttctgcgaactaacagg-3′ and reverse: 5′-gcaccactggggtaaggttt-3′; for *β-actin* (TM: 60°C), forward: 5′-catgtacgttgctatccaggc-3′ and reverse: 5′-ctccttaatgtcacgcacgat-3′.

### Assay for SOD, GSHPx and catalase activity

SH-SY5Y cells were harvested and then lysed using cell lysis solution (Beyotime Institute of Biotechnology, Jiangsu, China). Total protein concentration was quantified using the BCA Protein Assay Kit (Thermo Fisher Scientific Inc., Boston, MA, USA). The activity of SOD was determined using the total SOD assay kit with WST-1. The activities of GSHPx and catalase were determined using the cellular glutathione peroxidase assay kit and catalase assay kit according to the manufacturers’ instructions.

### Statistical analysis

Each experiment was repeated at least three times and results were expressed as the mean ± standard deviation (SD). Analysis of variance was carried out using SPSS 11.0 (SPSS, Chicago, IL, USA). Values of *P* < 0.05 were considered statistically significant.

## Results

### Geniposide, an active ingredient of TLJN, rescues the viability of SH-SY5Y cells treated with formaldehyde

**Figure 2:**
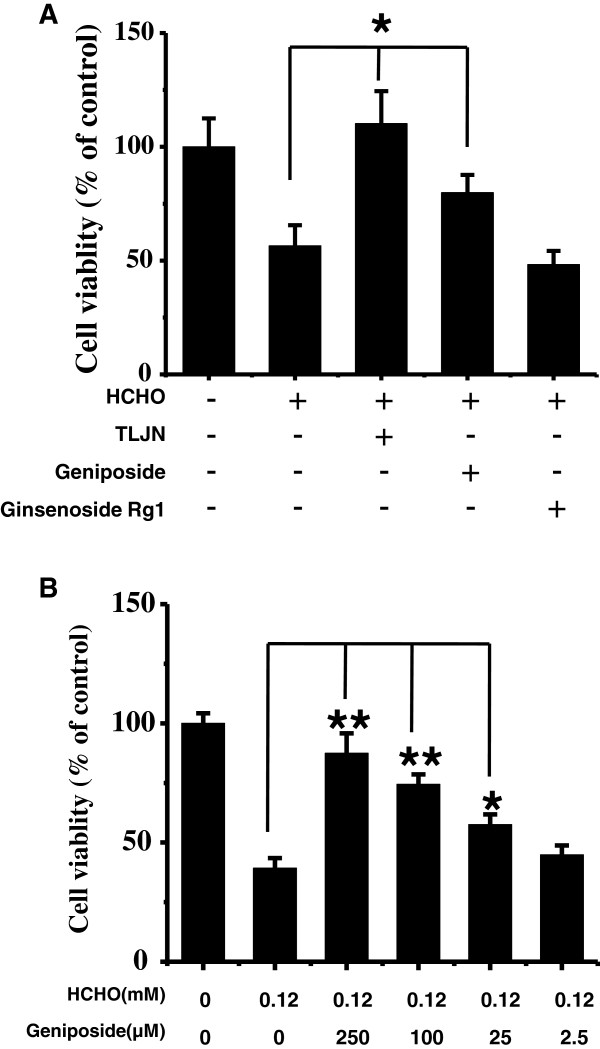
**TLJN and geniposide rescue SH-SY5Y cells following formaldehyde treatment.** SH-SY5Y cells were grown (1×10^4^) in 96-well plates overnight, and the culture medium was then replaced with serum-free DMEM in the presence of 0.12 mM formaldehyde, supplemented with TLJN (containing 100 μM geniposide and 10 μM ginsenoside Rg1), 100 μM geniposide, or 10 μM ginsenoside Rg1. Cell viability was evaluated using the cell counting kit-8 after a 24h incubation. Cells cultured without formaldehyde or drugs were used as a control **(A)**. Changes in SH-SY5Y cell viability following treatment with different concentrations of geniposide (250, 100, 25 and 2.5 μM) in the presence of formaldehyde for 24 h **(B)** were detected. Data are expressed as the mean ± SD from at least three independent experiments. *P < 0.05, **P < 0.01 vs. formaldehyde-only treated group.

TLJN preparation is clinically efficacious in the treatment of ischemic cerebral stroke and dementia [14,17]. In previous studies, we identified that TLJN has neuroprotective effects on ischemia/reperfusion-injured neurons and brain microvascular endothelial cells [14,18]. In this study, we investigated whether TLJN had a neuroprotective effect on formaldehyde-injured cells. SH-SY5Y cells (plated at 1×10^4^) were grown in serum-free DMEM in the presence of 0.12 mM formaldehyde supplemented with TLJN, geniposide or ginsenoside Rg1, to monitor changes in cell viability using CCK-8. As shown in Figure 2A, the relative viability of cells following formaldehyde treatment significantly decreased to 56.5% after a 24 h incubation when compared with the control (100% viability) in the absence of formaldehyde (*P* < 0.05). However, in the presence of TLJN, cell viability was maintained at control levels, indicating its ability to rescue SH-SY5Y cells from formaldehyde injury.

As described previously [17], the effective components of TLJN are geniposide and ginsenoside Rg1. First, geniposide was employed to protect the cells in the presence of formaldehyde. The viability of cells supplemented with both formaldehyde and geniposide was significantly higher than that with formaldehyde alone (*P* < 0.05, Figure 2A). Treatment with different concentrations of geniposide (2.5, 25, 100 and 250 μM) revealed that 25 μM (or higher concentrations) of geniposide significantly elevated cell viability from 39.3% to 57.6% (*P* < 0.05, Figure 2B). Second, the protective effect of ginsenoside Rg1 on cells was tested under the same conditions. In contrast, ginsenoside Rg1 could not significantly rescue the viability of cells following formaldehyde treatment (Figure 2A).

### Geniposide rescues the morphology of SH-SY5Y cells in the presence of formaldehyde

**Figure 3:**
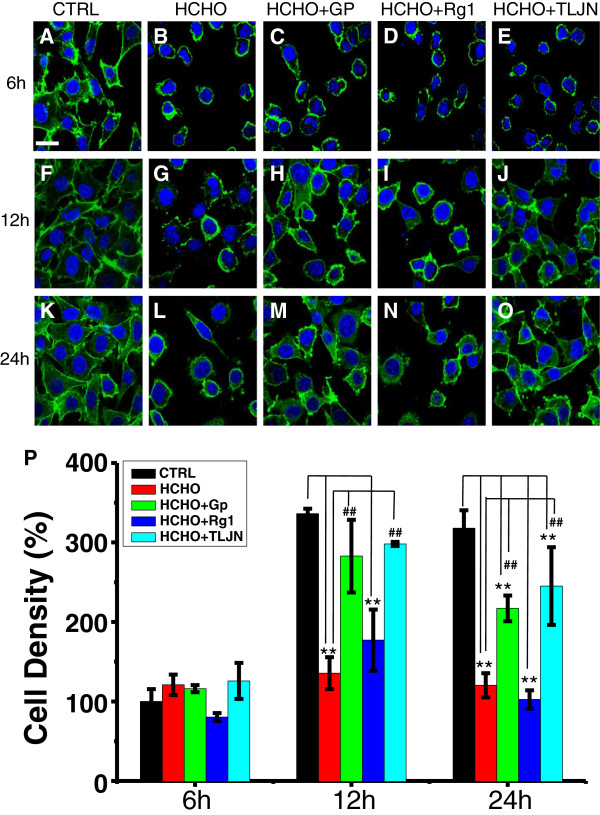
**TLJN and geniposide have a neuroprotective effect on SH-SY5Y cells exposed to formaldehyde.** Conditions were set as per Figure 2, except for the observation of nuclei (Hoechst, blue) and F-actin (Phalloidin, green). SH-SY5Y cells were incubated with 0.12 mM formaldehyde in the presence of 100 μM geniposide **(C**, **H**, **M**), 10 μM geniposide Rg1 **(D**, **I**, **N)**, or TLJN containing 100 μM of geniposide and 10 μM of ginsenoside Rg1 **(B**, **G**, **L)** at different time intervals (6, 12 and 24 h). Cells treated with formaldehyde alone **(E**, **J**, **O)** and no treatment **(A**, **F**, **K)** were used as controls. Scale bars = 20 μM. Cell density assays of SH-SY5Y cells at different time intervals (6, 12, and 24 h) **(P)**. Ctrl, control; FA, formaldehyde; Gp, geniposide; Rg1, ginsenoside Rg1. Data are expressed as the mean ± SD from three independent experiments.

Changes in cell morphology were also observed by laser scanning confocal microscopy at different time intervals following incubation (Figure 3). As shown in Figure 3E, SH-SY5Y cells treated with formaldehyde for 6 h shrank considerably when compared with the control (Figure 3A). This adverse effect of formaldehyde on cell growth was also observed at 12 and 24 h (Figure 3J and 3O). Cell morphological changes at 6 h were not significantly rescued in the presence of TLJN, geniposide or ginsenoside Rg1 when compared with formaldehyde treatment alone (Figure 3B, C, and D), suggesting that they did not exert a significant protective effect on cell growth at this early time point. Surprisingly, when cells were left for 12 h, the shape of SH-SY5Y cells incubated with formaldehyde plus TLJN appeared to be much healthier than the formaldehyde-treated only group (Figure 3G to 3J). A similar effect was found in cells supplemented with geniposide (Figure 3H). However, there was no significant difference between formaldehyde treatment and formaldehyde plus ginsenoside Rg1 treatment (Figure 3J to 3I). These results indicate that geniposide is the key component of TLJN that protects cells from the adverse effects of formaldehyde.

The numbers of surviving cells were also monitored in our study (Figure 3P). Formaldehyde treatment significantly decreased cell number at 12 and 24 h, revealing its neurotoxic effect (*P* < 0.01). TLJN and geniposide significantly rescued the number of SH-SY5Y cells treated with formaldehyde at both 12 and 24 h (*P* < 0.01), indicating that geniposide but not ginsenoside Rg1 is able to rescue the viability of formaldehyde-injured SH-SY5Y cells under our experimental conditions. The protective effect of ginsenoside Rg1 on cellular morphology and cell number could not be detected.

**Figure 4:**
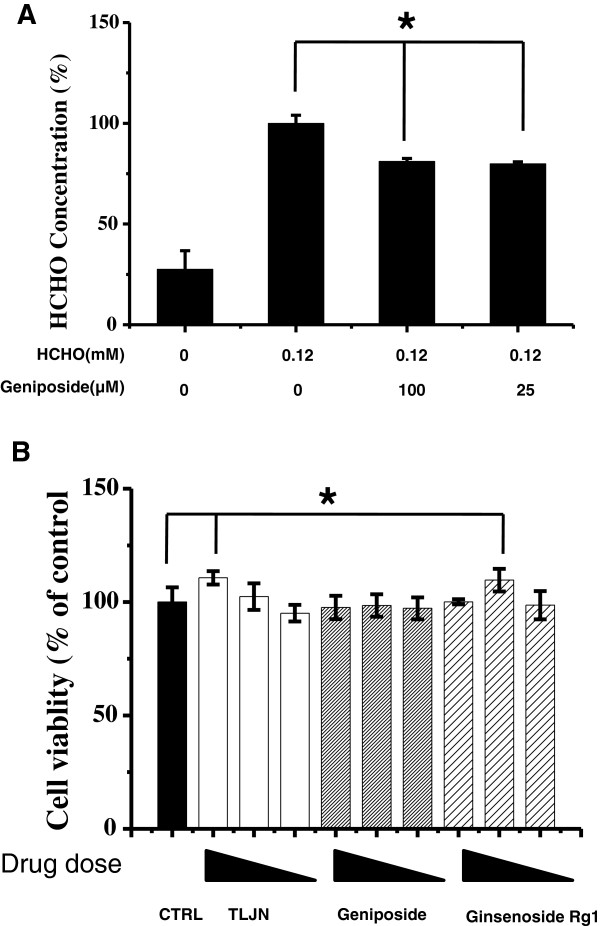
**Changes in formaldehyde levels in culture medium and cell viability in the presence of geniposide.** Decreases in formaldehyde concentrations in the medium of cells treated with geniposide (100 μM and 25 μM) for 24 h were detected by HPLC when compared with the formaldehyde-only group (control; **A**). Changes in SH-SY5Y cell viability in the presence of TLJN, geniposide, or ginsenoside Rg1 at different concentrations (250 μM, 100 μM and 25 μM; **B**). Data are expressed as the mean ± SD from at least three independent experiments. *P < 0.05, **P < 0.01 vs. the group treated with formaldehyde alone.

We were next concerned with why geniposide can rescue formaldehyde-treated SH-SY5Y cells. First, HPLC was used to measure changes in the concentrations of formaldehyde in the cell culture medium. The results showed that the residual content of formaldehyde decreased by 20% in the presence of 25 μM geniposide (*P* < 0.05, Figure 4A). We also confirmed that at the concentrations used in the experiments, geniposide, ginsenoside Rg1, and TLJN showed no cytotoxicity to SH-SY5Y cells (Figure 4B). Overall, these results suggest that geniposide rescues formaldehyde-treated cells at least through its capability of decreasing formaldehyde levels. Thus, we next explored the mechanism of this protective effect of geniposide on SH-SY5Y cells.

### Protection of SH-SY5Y cells is not caused by a direct interaction of geniposide with formaldehyde in the culture medium

**Figure 5:**
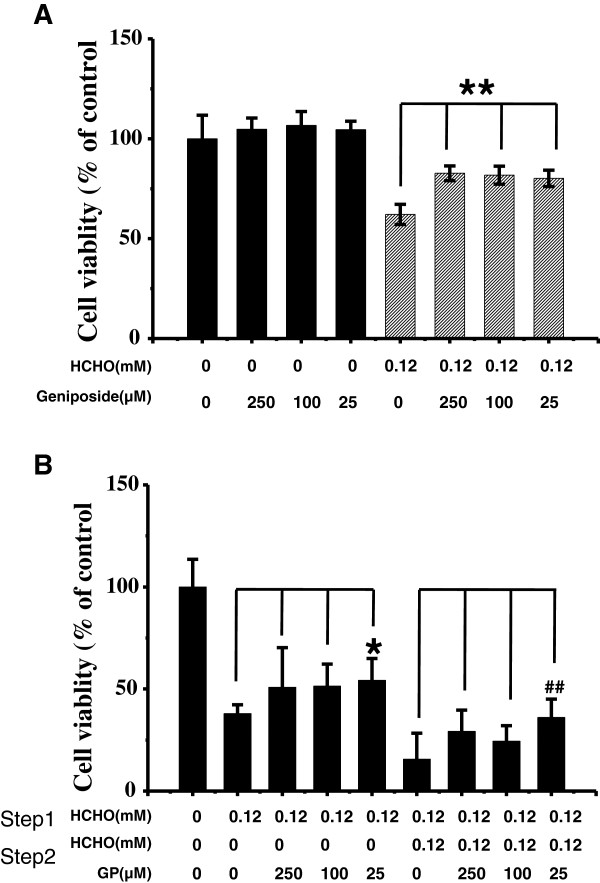
**Geniposide protects SH-SY5Y cells from formaldehyde injury.** Conditions for the treatment of cells were as described in Figure 2, except that SH-SY5Y cells were first incubated with different concentrations of geniposide overnight, followed by treatment with formaldehyde for 12 h. The viability of cells was evaluated using the cell counting kit-8 **(A)**. By contrast, cells were first exposed to formaldehyde overnight (conditions referred to in Figure 2), and then the medium was replaced with geniposide or both geniposide and formaldehyde for 24 h, followed by measurement of cell vitality **(B)**. Gp, geniposide. Data are expressed as the mean ± SD from three independent experiments. *P < 0.05, vs. the group treated with formaldehyde alone.

Because both geniposide and formaldehyde were added to SH-SY5Y cells at the same time, and it was shown that geniposide decreased cytotoxicity following formaldehyde treatment, we could not exclude the possibility that geniposide directly interacted with formaldehyde and then rescued the viability of cells. Therefore, in the following experiment we added geniposide to the cell culture medium before cells were treated with formaldehyde. Geniposide was removed after 12 h incubation with SH-SY5Y cells and then geniposide-treated cells were incubated with 0.12 mM formaldehyde for another 12 h. As shown by the CCK-8 assay (Figure 5A), cell viability of the geniposide-pretreated group was significantly higher (*P* < 0.01) than the formaldehyde alone treated group. Geniposide (25 μM) increased the viability of cells from 62.1% to 80.2%, similar to that of 100 and 250 μM geniposide.

On the other hand, we treated cells with 0.12 mM formaldehyde for 6 h first, and then incubated cells with geniposide or both geniposide and formaldehyde for 24 h. Results from the CCK-8 assay demonstrated that geniposide could improve viability following formaldehyde injury under either condition (Figure 5B). The addition of 25 μM geniposide to formaldehyde-injured cells increased their viability from 37.9% to 54.2% (*P* < 0.05). Viability increased from 15.6% to 36.1% in cells pretreated with formaldehyde for 6 h and in cells exposed to formaldehyde for 24 h following addition of geniposide (*P* < 0.01). This demonstrates that protection of SH-SY5Y cells by geniposide is not because of a direct interaction of geniposide with formaldehyde, but because of the activation of related cellular pathways.

### Geniposide suppresses apoptosis of SH-SY5Y cells in the presence of formaldehyde

**Figure 6:**
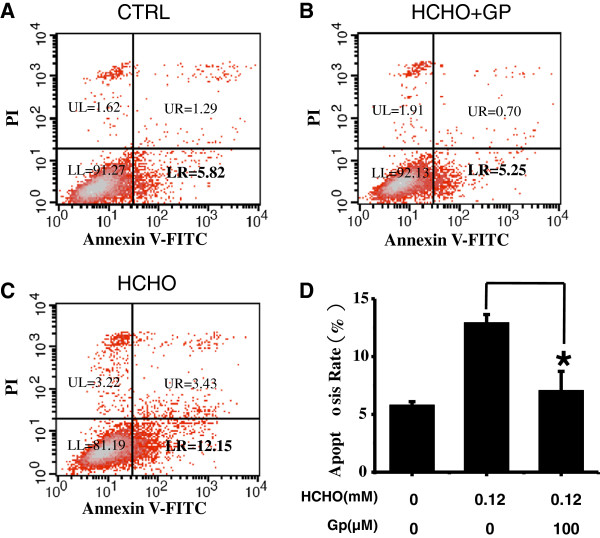
**Geniposide suppresses apoptosis of SH-SY5Y cells following formaldehyde treatment.** SH-SY5Y cells **(A)** were treated with 0.12 mM formaldehyde in the presence **(B**) or absence of 100 μM geniposide **(C)** for 24 h, and then the rate of apoptosis was detected by flow cytometry. Geniposide suppressed SH-SY5Y cell apoptosis following formaldehyde treatment as indicated. The percentages of apoptotic SH-SY5Y cells **(D)** were analyzed. Data are expressed as the mean ± SD from three independent experiments. *P < 0.05, vs. the group incubated with formaldehyde alone. The figures **(A**,**B**,**C)** are representative of three independent experiments.

To determine how geniposide protects cells from formaldehyde toxicity, we first monitored the rate of apoptosis after treatment. SH-SY5Y cells (plated at 1×10^6^) were grown in 60 mm plates overnight. Culture medium was then replaced with serum-free DMEM containing 0.12 mM formaldehyde supplemented with 25 or 100 μM geniposide for 24 h. Flow cytometry revealed that 5.82% of non-treated SH-SY5Y cells were apoptotic (Figure 6A). Incubation with 100 μM geniposide (Figure 6B) could suppress formaldehyde-induced apoptosis of SH-SY5Y cells with the percentage of apoptotic cells decreasing from 12.15% to 5.25% when compared with formaldehyde-only treated cells (Figure 6C), indicating the inhibitory effect of geniposide on apoptosis.

### Geniposide regulates apoptotic-related gene expression

**Figure 7:**
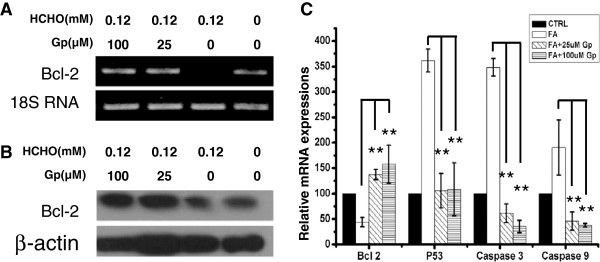
**Geniposide regulates apoptotic-related genes expressions.** Geniposide was able to regulate not only mRNA levels **(A)** but also protein levels of *Bcl-2***(B)** to antagonize formaldehyde-induced SH-SY5Y cell impairment. SH-SY5Y cells were incubated with formaldehyde and geniposide for 24 h. The expression of *Bcl-2*, *P53*, *caspase 3* and *caspase 9* mRNA were measured by quantitative real time PCR **(C)**. Treatment conditions were as described in Figure 2. Cells treated with formaldehyde alone and those with no treatment were used as positive and negative controls, respectively. Gp, geniposide. Data are expressed as the mean ± SD from three independent experiments. *P < 0.05, vs. the group treated with formaldehyde alone. The Gel electrophoresis is representative of three independent experiments.

Bcl-2 is the founding member of the *Bcl-2* family of apoptosis regulator proteins [19-21]. Thus, we investigated the changes in *Bcl-2* mRNA and protein expression. Our results showed that treatment of SH-SY5Y cells with formaldehyde induced a robust decrease in both *Bcl-2* mRNA and protein levels, followed by an increase in apoptotic cell death when cells were incubated with formaldehyde. However, treatment with different concentrations of geniposide (25 μM and 100 μM) was able to rescue levels of both *Bcl-2* mRNA (Figure 7A) and protein (Figure 7B) in the presence of formaldehyde. The expression of *Bcl-2* mRNA decreased by 56.1% after exposure to formaldehyde, but treatment with geniposide markedly increased the expression of *Bcl-2* from 213.2% (25 μM geniposide) to 259.3% (100 μM geniposide). We also measured the expressions of *P53*, *caspase 3* and *caspase 9* mRNA by quantitative real time PCR. P53 is a key modulator of the cellular stress response, and activation of *P53* can trigger apoptosis in many cell types including neurons [22]. Caspases play essential roles in apoptosis and caspase 3 is a key executer of apoptosis, whose activation is mediated by the initiator caspases, such as caspase 9 [23]. The expression of *P53*, *caspase 3* and *caspase 9* mRNA increased sharply by 261.5%, 248.2% and 90.5%, respectively, in the formaldehyde-treated group. In the geniposide-treated group, the expression of these genes decreased markedly by 70.7% (*P53*), 82.3% (*caspase 3)* and 75.8% (*caspase 9)* after 25 μM geniposide treatment and by 70.0% (*P53*), 89.8% (*caspase 3)* and 80.1% (*caspase 9)* after 100 μM geniposide treatment. These results suggest that *Bcl-2*, *P53*, *caspase 3* and *caspase 9* are involved in the protective effect of geniposide following formaldehyde injury of SH-SY5Y cells.

### Geniposide increases the activity of SOD and GSHPx

**Figure 8:**
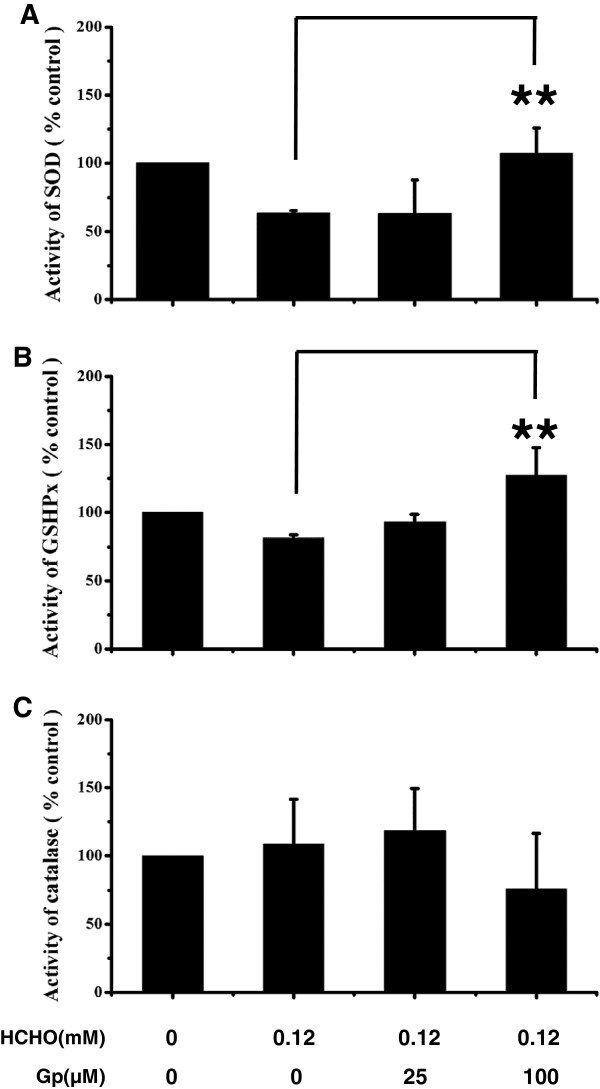
**Effect of geniposide on the activities of SOD, GSHPx and catalase in formaldehyde-injured SH-SY5Y cells.** After treatment of cells with different concentrations of geniposide (25, 100 μM) and 0.12 mM formaldehyde for 24 h. SOD activity **(A)**, GSHPx activity **(B)** and catalase activity **(C)** were measured using assay kits and a microplate reader. Cells treated with formaldehyde alone and those with no treatment were used as positive and negative controls, respectively. Gp, geniposide. The data are represented as the mean ± SD from three independent experiments. *P < 0.05 and **P < 0.01 vs. group treated with formaldehyde alone.

The generation of excess levels of reactive oxygen species (ROS) is important for the activation of apoptosis. SOD, GSHPx and catalase play a critical antioxidative role in tissues and organs [24]. Therefore, we next tested the activity of SOD, GSHPx and catalase in cells. As shown in Figure 8A and 8B, after exposure to formaldehyde, the activity of SOD and GSHPx decreased significantly (*P* < 0.05) when compared with the control. In contrast, treatment with 100 μM geniposide markedly increased the activity of SOD (from 63.7% to 107.2%, *P* < 0.01) and GSHPx (from 81.5% to 127.5%, *P* < 0.01) when compared with DMSO-incubated cells under our experimental conditions. The activity of catalase (Figure 8C) did not differ among the four experimental groups. This result demonstrates that geniposide improves the activity of SOD and GSHPx leading to suppression of apoptosis of SH-SY5Y cells in the presence of formaldehyde.

## Discussion

An increase in the concentration of endogenous formaldehyde (in blood and urine) has been found to correlate with an increase in age [7]. The concentration of hippocampal formaldehyde in AD patients is significantly higher than those of healthy seniors [7,25]. There is a certain amount of formaldehyde existing in the body, which is produced from several sources, such as the reaction of malondialdehyde with protein [20] and the methylation of DNA [5,26]. The cytotoxic mechanisms of formaldehyde have been studied in several experimental models. Formaldehyde is extremely reactive, cross-linking with proteins and with single stranded DNA, causing cellular dysfunction and even apoptosis [27]. Excess formaldehyde can also lead to endoplasmic reticulum stress and oxidative stress [28], which has been termed ‘formaldehyde stress’ [5]. Formaldehyde can also induce toxicity in neurons and astrocytes by multiple means, and in astrocytes, decreasing glutamate transporter expression, and inhibit glutamate uptake [29]. Moreover, formaldehyde is neurotoxic, and chronic overdosing increases the risk of AD and other related neurological disorders [30].

In our study, we found that TLJN could attenuate formaldehyde-induced neurotoxicity in cultured SH-SY5Y cells. Geniposide and ginsenoside Rg1 are the two main ingredients of TLJN [14]. In a previous study, we have shown that geniposide has a significant neuroprotective effect by blocking the progressive cascade that leads to cerebral ischemic injury and improving the expression of neurotrophic factors [13]. Moreover, ginsenoside Rg1 significantly stimulates neurite outgrowth of PC12 cells in the absence of nerve growth factor [31]. The present study indicates that geniposide is also able to attenuate formaldehyde-induced neurotoxicity, but that ginsenoside Rg1 is ineffective under these conditions. TLJN was more effective in neuroprotection than geniposide, exhibiting some integrated effects of geniposide and ginsenoside Rg1. Moreover, geniposide attenuated neurotoxicity in a dose-dependent pattern. Treatment with 25 μmol/L geniposide could increase cell viability from 39.3% to 57.6% (Figure 2B), with cell viability increasing markedly with increasing dosage.

In addition to cell viability, the morphology and cell number of SH-SY5Y cells following formaldehyde injury was rescued in the presence of geniposide and TLJN. While the protective effect of ginsenoside Rg1 on cellular morphology and cell number could not be detected. Furthermore, the concentration of formaldehyde in the cell culture medium significantly decreased after treatment with geniposide. Incubation with 25 μmol/L geniposide reduced the content of formaldehyde in the medium from 100% to 79.8%. This result indicates that there could be a direct interaction between geniposide and formaldehyde. However, this may also be because of the presence of several formaldehyde metabolic enzymes, such as alcohol dehydrogenase I, alcohol dehydrogenase III, formaldehyde dehydrogenase and aldehyde dehydrogenase 2 [1,5]. Therefore, the reason for the reduction in formaldehyde content may be because geniposide can upregulate the activity of formaldehyde metabolic enzymes or increase the metabolism of formaldehyde by healthy cells. We have tested whether geniposide could protect SH-SY5Y cells from formaldehyde injury following the addition of geniposide and formaldehyde to the cell culture medium in different orders. As shown in Figure 5, geniposide added to cultures before, at the same time, or after cells were exposed to formaldehyde resulted in an increase in cell viability. These results indicate that both possibilities, namely geniposide reacting with formaldehyde and protecting cells directly, are plausible. However, dose-dependent effects could not be observed under these experimental conditions. The lack of a dose-dependent effect suggests that 25 μM geniposide may already cause a maximal effect.

**Figure 9:**
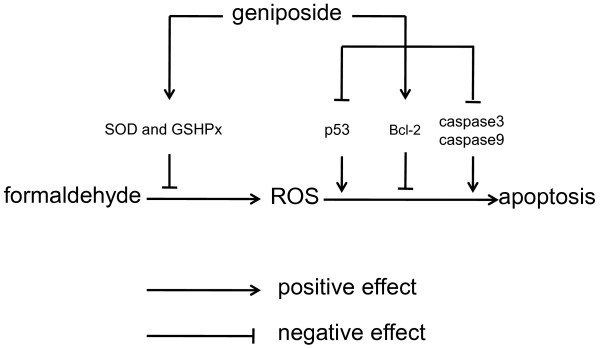
**A putative mechanism for the protective effect of geniposide on cells in the presence of formaldehyde.** Formaldehyde targets cell membranes and induces lipid peroxidation, inducing mitochondrial dysfunction, oxidative stress, and subsequent apoptosis. Reactive oxygen species (ROS), including singlet oxygen, hydrogen peroxide, superoxide anion and hydroxyl radicals, are important mediators of cellular injury, and play an important role in oxidative stress. ROS-initiated oxidative stress can be regulated by cell defense mechanisms, such as superoxide dismutase (SOD), which are a class of enzymes that catalyze the dismutation of superoxide and protect the cell from superoxide toxicity. *Bcl-2* is the founding member of the *Bcl-2* family of apoptosis regulator proteins, which tends to make cells more resistant to oxidative stress and apoptosis. P53 is a key modulator of the cellular stress response, and its activation can trigger apoptosis in many cell types including neurons. Caspase 3 is a key executer of apoptosis, whose activation is mediated by caspase 9. As shown in our results, geniposide is able to increase the activity of SOD and GSHPx and modulate the expression of *Bcl-2*, *P53*, *caspase 3* and *caspase 9*, thus inducing a neuroprotective effect on SH-SY5Y cells.

Geniposide, the active constituent of *Gardenia jasminoides*, which is widely used in Chinese traditional medicine, has been shown to have a neuroprotective effect against neuronal apoptosis induced by Aβ, CoCl_2_, and H_2_O_2_[19,32,33]. Our study showed that geniposide could also protect against neuronal apoptosis induced by formaldehyde. *Bcl-2* is an antiapoptotic oncoprotein that can promote cell survival [24]. *P53* is a key modulator of cellular stress responses, and its activation triggers apoptosis in many cell types including neurons [22]. Caspases play essential roles in apoptosis and *caspase 3* is a key executer of apoptosis, whose activation is mediated by the initiator caspases, such as *caspase 9*[23]. Herein, geniposide inhibited cell apoptosis through modulation of *Bcl-2*, *P53*, *caspase 3* and *caspase 9* mRNA levels. Oxidative stress is one of the major events that occur during neurological diseases such as AD [1]. In addition, oxidative stress is one of the main mechanisms for inducing apoptotic cell death, in which levels of ROS are upregulated [19]. Cells are susceptible to oxidative stress-induced apoptosis when levels of intracellular antioxidants are downregulated. SOD, GSHPx and catalase are important antioxidant defenses in nearly all cells exposed to oxygen free radicals. In our study, the activity of SOD and GSHPx in SH-SY5Y cells decreased significantly when exposed to formaldehyde. However, treatment with 100 μmol/L geniposide increased the activity of SOD from 63.7% (formaldehyde alone) to 107.2% and that of GSHPx from 81.5% to 127.5% (*P* < 0.01). Therefore, we propose that geniposide inhibits apoptotic cell death following formaldehyde injury by increasing the antioxidative and antiapoptotic capacity of cells (Figure 9).

## Conclusions

In the presence of TLJN, the viability of formaldehyde-treated SH-SY5Y cells remarkably recovered. In addition, the morphology of cells was rescued by TLJN and geniposide, an effective ingredient of TLJN. The activity of intracellular antioxidants (SOD and GSHPx), as well as mRNA and protein levels of the antiapoptotic gene *Bcl-2* were upregulated after the addition of geniposide. The expression of the apoptotic-related gene - *P53*, apoptotic executer - *caspase 3* and apoptotic initiator - *caspase 9* were downregulated after geniposide treatment. Our results indicate that geniposide can protect SH-SY5Y cells against formaldehyde stress by modulating the expressions of *Bcl-2*, *P53*, *caspase 3* and *caspase 9,* and increasing the activity of intracellular SOD and GSHPx.

## Abbreviations

AD: Alzheimer’s disease; TLJN: TongLuoJiuNao; TCM: Traditional Chinese medicine; GLP-1R: Glucagon-like peptide 1 receptor; SOD: Superoxide dismutase; DMEM: Dulbecco’s modified eagle medium; SD: Standard deviation; ROS: Reactive oxygen species; GSHPx: Glutathione peroxidase

## Competing interests

The authors declare that they have no competing interests.

## Authors’ contributions

PS, JC and MS performed the studies and analyzed the data. JL, WM and KL evaluated the data and performed the analysis. YM, JL, FW and YL conceived the study together and helped draft the manuscript. RH and QH conceived the project, and wrote and revised the manuscript. All authors have read and approved the final manuscript.

## Pre-publication history

The pre-publication history for this paper can be accessed here:

http://www.biomedcentral.com/1472-6882/13/152/prepub
